# Preparation and Pharmacokinetic Characterization of an Anti-Virulence Compound Nanosuspensions

**DOI:** 10.3390/pharmaceutics13101586

**Published:** 2021-09-29

**Authors:** Nan Wang, Feng Qi, Xiaolong He, Honglan Shi, David W. Anderson, Hao Li, Hongmin Sun

**Affiliations:** 1 Department of Mechanical and Aerospace Engineering, University of Missouri, Columbia, MO 65211, USA; nwang2019@sina.com (N.W.); gielcheen@gmail.com (F.Q.); 2 Department of Chemistry, Missouri University of Science and Technology, Rolla, MO 65409, USA; xh36z@mst.edu (X.H.); honglan@mst.edu (H.S.); 3 Ivogen Inc. (Subsidiary of Nanova, Inc.), Columbia, MO 65203, USA; AndersonDavid@nanovamed.com; 4 Department of Medicine, Division of Cardiovascular Medicine, University of Missouri, Columbia, MO 65212, USA

**Keywords:** biofilm, anti-virulence, wound infection, nanosuspension, pharmacokinetic

## Abstract

Antibiotic resistance has become a worldwide public health threat due to the rapid evolution and spread of antibiotic-resistant bacteria. CCG-211790 is a novel anti-virulence compound that does not kill bacteria but could ameliorate human diseases by inhibiting expression of virulence factors, thereby applying less selection pressure for antibiotic resistance. However, its potential clinical use is restricted because of its poor aqueous solubility, resulting in formulation challenges. Nanosuspension technology is an effective way to circumvent this problem. Nanosuspensions of CCG-211790 with two different particle sizes, NanoA (315 ± 6 nm) and NanoB (915 ± 24 nm), were prepared using an antisolvent precipitation-ultrasonication method with Tween 80 as the stabilizer. Particle and pharmacokinetics (PK) of CCG-211790 nanosuspensions were characterized. Both NanoA and NanoB demonstrated remarkable increases in dissolution rate compared with the bulk compound. The PK parameters of NanoA were comparable to those of CCG-211790 solution formulation in intravenous or oral administration, suggesting that CCG-211790 nanosuspensions with smaller particle size improved oral bioavailability and drug exposure compared to traditional formulations of drug candidates.

## 1. Introduction

Antibiotic resistance has become a major public health threat worldwide [1,2]. Conventional antibiotics select resistant bacterial pathogens by eliminating drug-sensitive competitors while leaving drug-resistant bacteria to multiply [3]. The increasing occurrence of antibiotic-resistant strains calls for new strategies that incur less selective pressure [4]. Anti-virulence agents that ameliorate human diseases by reducing the expression of virulence factors rather than killing or inhibiting the growth of bacterial pathogens provide an alternative approach to treat infections by antibiotic resistant pathogens [5]. We have identified a chemical series of low-molecular-weight compounds that target the expression of virulence factors of *Streptococcus pyogenes* and *Staphylococcus aureus* [6,7,8]. CCG-211790, with the IUPAC name 9-methoxy-3,5,5-trimethyl-2-((2,2,2-trifluoroethyl)thio)-5,6-dihydrobenzo[h]quinazolin-4(3H)-one, is one of the lead compounds [9]. Previous biological studies demonstrated that CCG-211790 inhibited the biofilm formation of methicillin-resistant *Staphylococcus aureus* (MRSA) [9], a biofilm forming nosocomial pathogen and one of the most notorious multi-drug resistant bacteria that causes skin and soft tissue infections (SSTIs) [10,11,12,13,14]. Bacterial biofilm formation and drug resistance make the infected wounds recalcitrant to antibiotic treatment and could lead to systemic life-threatening infections and sepsis [14]. Biofilm formation is a key pathology of non-healing wounds [15,16]. Bacteria in biofilms are much more tolerant to antimicrobial agents and disinfectants [17,18,19], as well as host defense mechanisms [20,21]. Potent antibiotics or their combinations are usually used to treat complicated SSTIs caused by biofilm forming and multi-drug resistant bacteria, but their use may be associated with life-threatening toxicities such as nephrotoxicity and hepatotoxicity [22,23,24]. Thus there is a great unmet clinical need for novel anti-biofilm agents to treat infections associated with chronical wounds [14]. 

CCG-211790 and its analogs inhibit MRSA virulence and biofilm formation, making them attractive candidates for monotherapy or complementing current antibiotics for treating wound infections [6,9]. However, the clinical use of CCG-211790 is restricted by its low aqueous solubility (less than 50 ng/mL), leading to formulation challenges such as erratic absorption and poor oral bioavailability for oral formulations [25]. A topical formulation of CCG-211790 was developed for application in wound treatment [9], yet there are many challenges to treating skin infections locally. Wet wound environment and enzymes within the wound tissue reduce the efficacy of the topically applied drugs [26]. As a result, it is necessary to further improve the pharmacokinetic properties of this novel anti-biofilm compound to enhance its potential for treating wound infections. Absorption of orally administrated solid dosage forms of drugs into the systemic circulation involves three factors. Dosage form disintegration, drug dissolution, and drug permeation across intestinal cell membranes into the systemic circulation all affect drug absorption [27,28,29,30]. For poorly water-soluble drugs, especially the BCS II compounds, drug absorption is often limited by the drug dissolution rate from the dosage forms (k_d_<<k_a_). The maximum drug plasma concentration (C_max_) and time to reach the C_max_ for this type of poorly water-soluble drug is dictated by the dissolution rate of the drug from the dosage form. Additionally, the fraction of drug absorbed will be affected by the drug dissolution rate if the time required for complete dissolution is longer than the transit time of the dosage form at the drug absorptive sites. These properties affect the overall bioavailability of drugs and their overall effectiveness [27].

In this study, nanosuspension technology that reduces the particle size of drug crystals to submicron scale was employed for the development of CCG-211790 formulations. Nanosuspension is defined as the colloidal dispersion of nanosized drug particles (100–1000 nm) in an aqueous vehicle such as water, aqueous solution or non-aqueous media stabilized with surfactants or polymeric stabilizers [28]. Nanosuspension technology is a promising approach for addressing poor-solubility problems and has many advantages including: (1) enhanced dissolution rate and oral bioavailability compared with raw drugs or coarse suspensions [31,32]; (2) high drug payload (e.g., 20–30% concentrated nanosuspensions produced via wet milling method, and up to 90% nanocrystal powder-loaded tablets) is available with benefits to high-dosing situations [33,34]; (3) nanoparticles contain almost 100% pure drug with low content of irritant excipients (e.g., surfactant and/or co-solvents) that reduces excipient-induced toxicity and improves drug safety [35]; (4) diverse dosage forms such as tablets, pellets, nanosuspensions, suppositories and hydrogels are available giving flexibility for various administration routes such as intravenous (IV) [36], intramuscular [37], oral [38], ocular [39], and nebulization [40]. 

In general, nanosuspensions can be produced either by bottom-up approaches consisting of dissolved drug molecules precipitated or crystallized from a supersaturated solution forming solid nanoparticles [41,42], or by top-down approaches where large particles are reduced into nanosized particles via comminution processes [43,44]. In this study, two CCG-211790 nanosuspension formulations with different particle sizes and morphologies, termed as NanoA (with z-average of 315 ± 6 nm) and NanoB (with z-average of 915 ± 24 nm), respectively, were prepared using antisolvent precipitation-ultrasonication method, which is a bottom-up approach. Particle size, morphology, stability, in vitro dissolution profiles, and in vivo pharmacokinetics (PK) of nanosuspensions were investigated. 

## 2. Materials and Methods

### 2.1. Materials

The synthesis of CCG-211790 has been described previously [9]. Tween^®^ 80 (polyoxyethylene-20-sorbitan monooleate), polyethylene glycol 400 (PEG 400) and dimethyl sulfoxide (DMSO) were purchased from Sigma-Aldrich (Saint Louis, MO, USA). Propylene glycol was purchased from MP Biomedicals LLC (Irvine, CA, USA). Acetonitrile and sodium dodecyl sulfate (SDS) were purchased from Fisher Scientific (Pittsburgh, PA, USA). Fractionated coconut oil was purchased from Piping Rock Health Products, LLC (Ronkonkoma, NY, USA). Ethanol was purchased from Decon Laboratories Inc. (Detroit, MI, USA). Liquid coconut oil was purchased from Piping Rock Health Products, LLC (Ronkonkoma, NY, USA) and used as received. Deionized water was supplied from a Culligan reverse osmosis filtration system (Rosemont, IL, USA). All chemicals except coconut oil were of analytical or high-performance liquid chromatography (HPLC) grade. 

### 2.2. Preparation of CCG-211790 Nanosuspensions 

CCG-211790 nanosuspensions with two different particle sizes were prepared by an antisolvent precipitation-ultrasonication method and were named NanoA (315 ± 6 nm) and NanoB (915 ± 24 nm), depending on the hydrodynamic particle size obtained by dynamic light scattering (DLS). Briefly, NanoA was obtained by injecting 0.5 mL of CCG-211790 solution (15 mg/mL CCG-211790 in DMSO) into 5 mL of Tween 80 solution (0.005% *w*/*v* in water) under magnetic stirring (PC-420D stirring hot plate, Corning Inc., Corning, NY, USA) at 1000 rpm, continuing with intense sonication for 10 min in an ultrasonic bath (Bransonic B221, Emerson Electric, St. Louis, MO, USA) filled with 600 mL of water. During this process, mechanical stirring at 600 rpm was applied by an overhead mixer (OS40-S, Scilogex LLC, Rocky Hill, CT, USA). Similarly, NanoB was obtained by injecting 0.5 mL of CCG-211790 solution (15 mg/mL CCG-211790 in DMSO) into 5 mL of Tween 80 solution (0.02% *w*/*v* in water) under magnetic stirring at 500 rpm, continuing with intense sonication and mechanical stirring at 400 rpm for 10 min in the ultrasonic bath filled with 220 mL water. DMSO was then removed from suspensions by dialysis (Fisher brand regenerated cellulose membrane with 12,000 to 14,000 MW cutoff) against water. CCG-211790 concentration was adjusted to 1 mg/mL with water prior to use.

### 2.3. Dynamic Light Scattering (DLS)

The mean hydrodynamic particle size (z-average or z_avg_), polydispersity index (PDI) and zeta potential of CCG-211790 suspensions were determined by the Zetasizer Nano ZS (Malvern Panalytical, Malvern, United Kingdom). The measurements of hydrodynamic particle size and PDI were performed in deionized water at 25 °C [45]. PDI is a parameter evaluating the width of particle size distribution. Mean values and standard deviations were calculated.

### 2.4. Scanning Electron Microscopy 

The morphologies of freeze-dried CCG-211790 nanosuspensions and CCG-211790 bulk powder were observed using a scanning electron microscope (SEM) (FEI Company, Hillsboro, OR, USA). SEM samples of nanosuspensions were prepared by freeze-drying CCG-211790 nanosuspensions in a FreeZone 1 Liter Benchtop Freeze Dryer (Labconco Corporation, Kansas City, MO, USA) immediately after preparation. Briefly, suspensions (0.5 mL) were pre-frozen at −50 °C for 2 h and then were freeze-dried at −50 °C and 0.015 mbar for 48 h to yield dry powders. CCG-211790 bulk powder was used as received following synthesis. Each sample was fixed on an aluminum SEM stage with a conductive double-sided carbon tape. 

### 2.5. High-Performance Liquid Chromatography (HPLC) Analysis of Nanosuspensions

The concentrations of CCG-211790 nanosuspensions were measured using an Agilent series 1100 HPLC system (Agilent Technologies, Santa Clara, CA, USA) equipped with a diode array detector. Sample separation was carried out with a Kinetex C18 column (75 mm × 3 mm, particle size 2.6 µm, Phenomenex, Torrance, CA, USA). The mobile phase was acetonitrile-water (50:50, *v/v*), flow rate was set to 0.5 mL/min, and column temperature to 35 °C. The injection volume was 30 µL. The absorption wavelength was set at 255 nm. CCG-211790 nanoparticles were dissolved in the mobile phase for HPLC analysis. Due to the fact that the analyte concentration was high and no other matrix was present in the sample, the HPLC method performed very well with excellent calibration linearity, reproducibility, and spike recovery. During sample analysis, ongoing quality controls were performed including a reagent blank, a duplicate sample, and spike recovery samples for every 10 samples analyzed.

### 2.6. Stability of CCG-211790 Nanosuspensions

The representative batch of NanoA and NanoB nanosuspensions were stored at 4 °C or room temperature. Z-average, PDI and zeta potential were monitored by DLS over a period of six weeks. Due to the fact that NanoA was produced in 0.005% Tween 80 solution while NanoB was produced in 0.02% Tween 80 solution, both NanoA and NanoB nanosuspensions (0.2 mL) were diluted with 0.02% Tween 80 solution (2 mL) prior to each measurement to maintain consistent medium vehicle.

### 2.7. In Vitro Dissolution Study

The in vitro dissolution profiles of CCG-211790 bulk powder, NanoA and NanoB suspensions were examined. SDS is one of the most frequently used artificial surfactants in dissolution test to establish sink conditions for water-insoluble drugs. Therefore, a SDS solution (0.5% *w/v* in water) was used as the dissolution medium in this study. In each dissolution test, suspension or powder equivalent to 4 mg of CCG-211790 were directly dispersed in 500 mL dissolution medium under magnetic stirring at 75 rpm. The temperature of the dissolution medium was maintained at 37 ± 1 °C in a water bath. Sample aliquots (3 mL) were withdrawn at predetermined time points (5, 10, 20, 30, 45, and 60 min) and immediately replaced with 3 mL of fresh dissolution medium to readjust the volume to 500 mL. Undissolved particles were separated by centrifugation at 10,000× *g* for 5 min and the supernatant was collected for HPLC analysis. All experiments were run in triplicate. 

### 2.8. Pilot PK Study of CCG-211790 Powder Suspension

The pilot PK studies of CCG-211790 powder suspension formulations via intravenous administration and oral delivery were outsourced to Gateway Pharmacology Laboratories (Chesterfield, MO, USA). Four male Sprague-Dawley rats (180–200 g) were purchased from Harlan Laboratories (Indianapolis, IN, USA). All animals were acclimatized at a temperature of 22 ± 4 °C and a relative humidity of 30–70% under a 12 h light/dark condition (lights on at 6:00 AM and off at 6:00 PM). All animals were allowed free access to standard rodent chow (PicoLab Rodent Diet 20) and water.

A solution formulation of CCG-211790 to a concentration of 0.5 mg/mL was freshly prepared prior to dosing by mixing 30 μL stock solution (50 mg/mL in DMSO) into 2970 μL 0.9% saline. Two rats received the solution formulation 2.5 mg/kg CCG-211790 via tail vein injection at a dosing volume of 5 mL/kg with blood samples collected at 5, 15, and 30 min; 1, 3 (via retro-orbital bleeds) and 6 (via cardiac puncture) hours post-dose. Another two rats received 5 mg/kg CCG-211790 bulk powder dispersed in 0.5% *w/v* of carboxymethyl cellulose (CMC) solution via oral gavage at a dosing volume of 10 mL/kg. Blood samples were collected at 0.5, 1, 2, 4, 6 (via retro-orbital bleeds), and 8 (via cardiac puncture) hours post-dose. Plasma samples were analyzed by HPLC-MS/MS method. Analysis was performed using a Shimadzu HPLC system (Shimadzu Corporation, Columbia, MD, USA) equipped with a 4000Q Trap tandem mass spectrometer (AB Sciex, Foster City, CA, USA). The method was developed by tuning the instruments for the test article based on the molecular weight of the compound, following an industry standard fit-for-purpose approach/assay for pharmacokinetic/pharmacology studies where concentration versus time data were beneficial in understanding the exposures. A Kinetex C18 (2.1 × 50 mm, particle size 2.6 µm, Phenomenex, Torrance, CA, USA) was used for separation. Samples were eluted with a flow rate set to 0.4 mL/min under a gradient elution program with eluent A (ultra-pure water with 0.1% (*v/v*) formic acid) and eluent B (acetonitrile with 0.1% (*v/v*) formic acid). 

### 2.9. PK Studies of Nanosuspensions

PK studies of nanosuspensions via intravenous administration and oral administration were performed in male Sprague-Dawley rats (450 ± 32 g and 425 ± 19 g, respectively). All animals were purchased from Charles River Laboratories (Wilmington, MA, USA). The animals were acclimatized at a temperature of 25 ± 2 °C and a relative humidity of 75 ± 5% under natural light/dark condition for one week. The animals were kept under fasting overnight prior to the experiments. All experimental procedures were approved by the Animal Care and Use Committee (ACUC) of University of Missouri (Columbia, MO, USA).

For IV PK studies, Sprague-Dawley rats were randomly divided into three groups with six rats in each group. A solution formulation of CCG-211790 dissolved in a solution of ethanol (10%), propylene glycol (25%), PEG 400 (35%) and water (30%) to a concentration of 1 mg/mL, termed as PEG/PG solution, was prepared as the control. The PEG/PG solution, NanoA and NanoB suspensions were administrated to the three groups respectively at a dose of 2.5 mg/kg via bolus tail vein injection. Blood samples were collected in EDTA-coated tubes via femoral vein bleeding at predetermined time points (5, 15, and 30 min; 1 h, 2 h, and 4 h). Plasma was separated from the blood by centrifugation at 1000× *g* for 10 min and stored at −80 ℃ until analysis.

For oral PK studies, Sprague-Dawley rats were randomly divided into three groups with six rats in each group. A coconut oil solution formulation of CCG-211790 dissolved in fractionated coconut oil to a concentration of 1 mg/mL was prepared as the control. The coconut oil solution and NanoA and NanoB suspensions were administrated by oral gavage (10 mL/kg) at a dose of 5 mg/kg, respectively. Blood samples were collected in EDTA-coated tubes via femoral vein bleeding at predetermined time points (0.5, 1, 2, 4, 6, and 8 h post-dose). Plasma was separated from the blood by centrifugation at 1000× *g* for 10 min and stored at −80 °C until analysis. Plasma samples were analyzed by UPLC-MS/MS method.

### 2.10. UPLC-MS/MS Analysis of Plasma Samples

The concentration of CCG-211790 in plasma is low and a more sensitive UPLC-MS/MS method was developed to detect its concentration in plasma samples. A procedural calibration protocol was used for calibration standard preparation using a control plasma that did not contain CCG-211790 and internal standard. Different concentrations of calibration standard were added into the control plasma and processed through the same procedure as the plasma sample preparation. For standard and sample preparation, 30 μL plasma was mixed with 120 μL acetonitrile containing an internal standard tolbutamide followed by 30 min wait to completely precipitate the protein in the plasma, which was then centrifuged at 10,000× g for 10 min. The supernatant (100 μL) was transferred to an autosampler vial for UPLC-MS/MS analysis. UPLC-MS/MS analysis was performed using a Shimadzu UPLC system (Shimadzu Corporation, Columbia, MD, USA) equipped with a 4000Q Trap tandem mass spectrometer (AB Sciex, Foster City, CA, USA). A Gemini 3u C18 column (50 mm × 2 mm, particle size 3 µm, Phenomenex, Torrance, CA, USA) was used for separation. Samples were eluted with a flow rate set to 0.8 mL/min under a gradient elution program with eluent A (ultra-pure water with 0.1% (*v/v*) formic acid) and eluent B (acetonitrile with 0.1% (*v/v*) formic acid). The gradient was set as follows: 20 % eluent B maintained for 1 min followed by linear increase to 100% over 2 min and maintained at 100% eluent B for 3 min. The total runtime was 6 min. The sample injection volume was 10 µL. Column temperature was set to 30 °C. The 4000Q Trap spectrometer was operated under positive electrospray ionization (+ESI) mode and using multiple reaction monitoring (MRM) for quantitation. The quantification ion pair was 385.088 > 156 and confirmation ion pair was 385.088 > 73.1 for CCG-211790. The quantification ion pair used for internal standard was 271.108 > 90.9 and confirmation ion pair was 271.108 > 74. All the other parameters were optimized for the most sensitive ion transition for quantification. The method was validated prior to plasma sample analysis. The validation conditions included reproducibility, calibration linearity, detection limit, and spike recovery. The method quantification detection limit was 0.5 ng/mL. The calibration showed very good linearity (R^2^ > 0.99). Strict ongoing quality control tests were performed during all the sample analyses, including a quality control standard check, a reagent blank, a spike recovery, and a duplicate sample for every 10 samples analyzed to certify precisions and accuracies of the analyses.

### 2.11. Statistics

PK parameters were analyzed via non-compartmental analysis using PK solver 2.0 software, which is a freely available menu-driven add-in program for Microsoft Excel, for PK and pharmacodynamic (PD) data analysis (China Pharmaceutical University, Nanjing, China) [46]. Statistical differences were estimated via Student’s *t*-test with *p* < 0.05 as significant.

## 3. Results

### 3.1. Particle Size and Morphology

**Figure 1:**
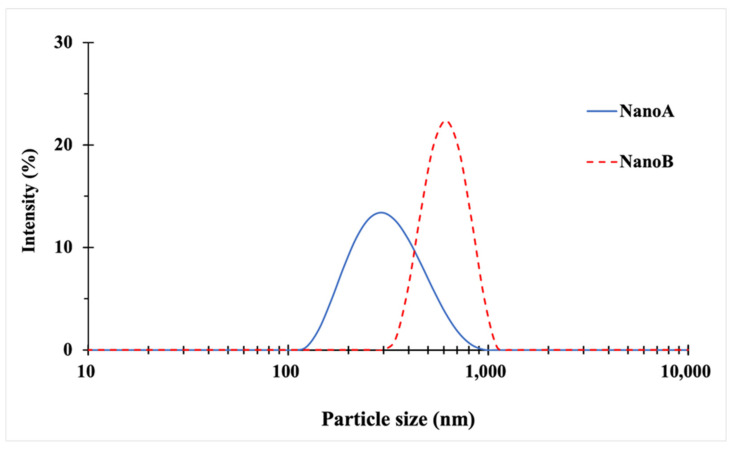
Hydrodynamic particle sizes of the representative batch of NanoA and NanoB suspension after the preparation obtained by DLS.

The hydrodynamic particle size and the z-average, of NanoA and NanoB suspensions were determined by DLS after the dialysis. It is worth noting that the hydrodynamic particle size is an apparent value equivalent to the diameter of a sphere having the same average diffusion coefficient as the particles being measured [47]. In this study, the preparation of NanoA samples (*n* = 21) demonstrated good reproducibility, while NanoB samples (*n* = 9) displayed wider particle size distribution. In general, the z-average values of NanoA ranged from 300–400 nm with a mean value of 317 ± 20 nm. Similarly, the z-average values of NanoB samples ranged from 680–950 nm with a mean value of 801 ± 110 nm. One representative batch of NanoA (315 ± 6 nm) and one representative batch of NanoB (915 ± 24 nm) were selected for the following stability study (Figure 1).

**Figure 2:**
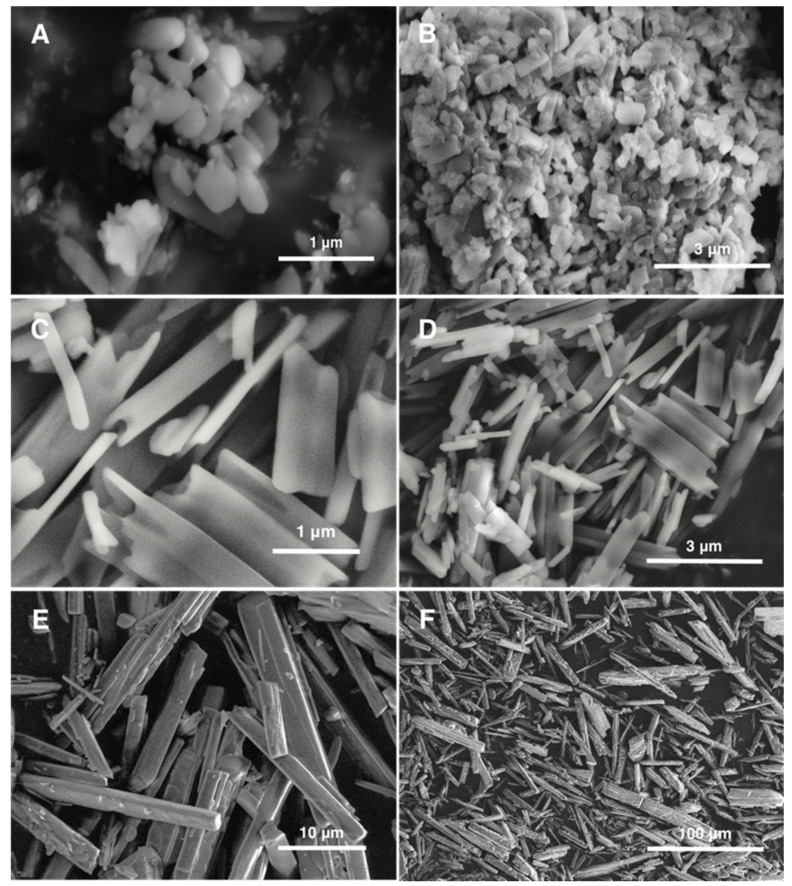
SEM images of NanoA (**A**,**B**), NanoB (**C**,**D**), and CCG-211790 bulk powder (**E**,**F**).

The morphologies of CCG-211790 bulk powder, NanoA and NanoB freeze-dried powders were examined by SEM. The majority of particles in NanoA were small particles with dimensions from 200–400 nm, while a few larger plate-like particles also existed with lengths typically from 500–1100 nm and widths less than 400 nm (Figure 2A,B). In contrast, plate-like and tube-like particles were produced in NanoB with lengths mostly around 0.5–3 μm, widths at the submicron-scale and thicknesses down to a hundred nanometers (Figure 2C,D). CCG-211790 bulk powder was more heterogeneous and consisted of a large number of relatively small particles with lengths up to twenty micrometers and diameters of a few micrometers. In addition, a few relatively large particles with lengths over fifty micrometers and diameters over twenty micrometers were observed, demonstrating the heterogeneity of the bulk powder (Figure 2E,F).

### 3.2. Storage Stability of Nanosuspensions

NanoA and NanoB suspensions were stored at 4 °C and room temperature, z-average, PDI and zeta potential were monitored by DLS for six weeks. Although DLS does not produce results directly related to the size of high aspect-ratio particles, it is still useful to roughly track the change of the particle size over time [48]. 

At both 4 °C and room temperature, there were no significant differences of z-average (315 ± 6 nm vs. 315 ± 3 nm, 315 ± 6 nm vs. 319 ± 5 nm), and PDI (0.18 ± 0.03 vs. 0.21 ± 0.01, 0.18 ± 0.03 vs. 0.20 ± 0.03) for NanoA suspension after 6 weeks, with similar results found for NanoB. Overall, both NanoA and NanoB were relatively stable within 6 weeks, (Table 1 and Table 2), probably because a minimum zeta potential of ±30 mV was sufficient to provide electrostatic barrier against particle-particle aggregation [49,50].

### 3.3. In Vitro Dissolution Studies

**Figure 3:**
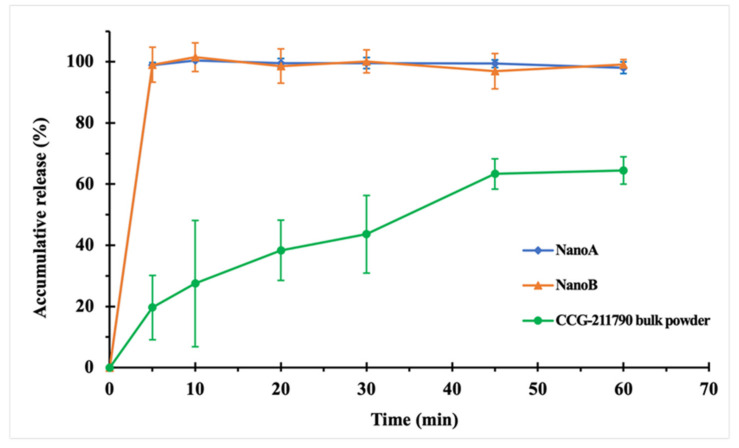
In Vitro dissolution profiles of CCG-211790 bulk powder and NanoA and NanoB suspensions in 0.5% SDS solution (*n* = 3). There were statistically significant differences between the dissolution rates of NanoA and NanoB and the dissolution rate of CCG-211790 bulk powder (*p* < 0.05), while there was no statistical difference between NanoA and NanoB (*p* > 0.05).

Dissolution profiles (Figure 3) showed significant increases in dissolution rates for NanoA and NanoB suspensions compared to CCG-211790 bulk powder. The drug releases for NanoA and NanoB suspensions were 99% at 5 min whereas bulk powder CCG-221790 release was 19.6% at 5 min. Bulk powder release was slow, reaching a maximum of 64.3% after 60 min.

### 3.4. Pilot PK Studies

**Figure 4:**
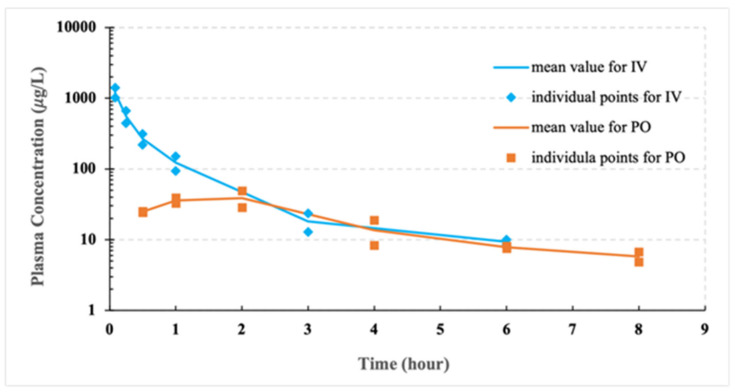
Plasma concentration–time curve for pilot study of CCG-211790 powder suspension (*n* = 2) and a DMSO/saline solution (*n* = 2) either following oral administration or intravenous administration in Sprague–Dawley rats at a dose of 5 mg/kg or 2.5 mg/kg, respectively.

The CCG-211790 plasma concentration-time curves and the PK parameters for pilot study are shown in Figure 4, Table 3 and Table 4 respectively. Bioavailability (F) is one of the principal pharmacokinetic properties of drugs defined as the fraction of an administered dose of unchanged drug that reaches systemic circulation [51]. The bioavailability of a drug given by the intravenous route is considered 100% (F = 1), and usually less than one if given by other routes; bioavailability close to 100% indicates complete absorption of the drug, which is desirable in the development of extra-vascular or oral formulations [52]. The bioavailability of an oral formulation is given by $F=\frac{AUC_{po}\cdot Dose_{IV}}{AUC_{IV}\cdot Dose_{PO}}\times 100\%$, where AUC is the area under the concentration-time curve, and Dose_IV_ and Dose_PO_ were 2.5 mg/kg and 5 mg/kg, respectively, for the pilot PK study.

The bulk compound had poor oral bioavailability at 13.4%, likely due to poor aqueous solubility of the compound [25].

### 3.5. PK Studies

**Figure 5:**
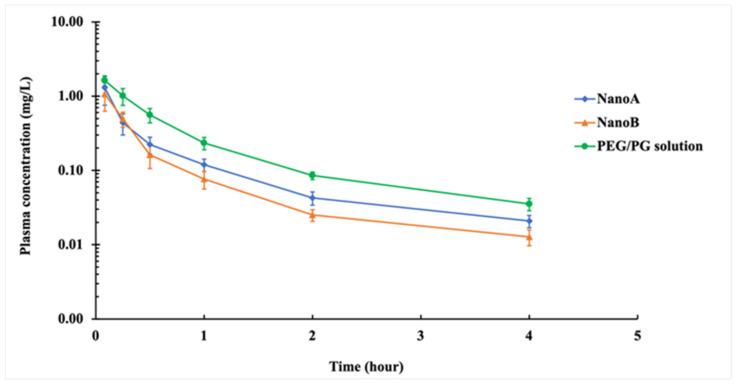
Plasma concentration–time curve of PEG/PG solution and NanoA and NanoB suspensions after intravenous administration in Sprague–Dawley rats at a dose of 2.5 mg/kg (*n* = 6).

The CCG-211790 plasma concentration-time curves and the PK parameters of PEG/PG solution (control), NanoA and NanoB suspensions following intravenous administration in Sprague-Dawley rats at a dose of 2.5 mg/kg are shown in Figure 5 and Table 5, respectively.

NanoA and NanoB showed trends toward lower C_max_ (1.31 ± 0.55 mg L^−1^, 1.07 ± 0.44 mg L^−1^), as compared to PEG/PG solution (1.63 ± 0.25 mg L^−1^). The clearance (CL) of NanoA (4.21 ± 1.28 L h^−1^ kg^−1^) and NanoB (5.28 ± 1.01 L h^−1^ kg^−1^) were higher than that of PEG/PG solution (2.28 ± 0.27 L h^−1^ kg^−1^). The AUC of NanoA (0.64 ± 0.19 mg L^−1^ h) and NanoB (0.49 ± 0.12 mg L^−1^ h) were lower than that of PEG/PG solution (1.11 ± 0.14 mg L^−1^ h). The V_z_s of the NanoA (6.44 ± 1.84 L kg^−1^) and NanoB (8.37 ± 3.29 L kg^−1^) were higher than PEG/PG solution (3.66 ± 0.93 L kg^−1^).

**Figure 6:**
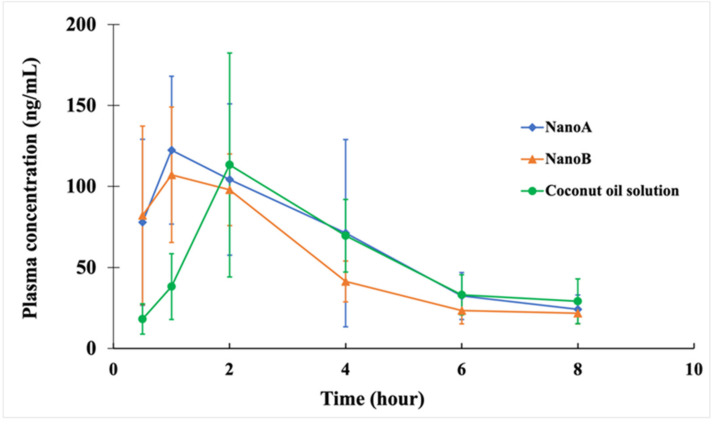
Plasma concentration–time curve of the coconut oil solution (*n* = 6) and NanoA and NanoB suspensions (*n* = 6) after oral administration in Sprague–Dawley rats at a dose of 5 mg/kg.

The CCG-211790 plasma concentration-time curves and the PK parameters of coconut oil solution, NanoA and NanoB suspensions following oral administration in Sprague-Dawley rats at a dose of 5 mg/kg are shown in Figure 6 and Table 6, respectively.

The C_max_ values for NanoA (0.13 ± 0.05 mg L^−1^), NanoB (0.12 ± 0.03 mg L^−1^), and coconut oil solution (0.12 ± 0.07 mg L^−1^) were nearly the same. The T_max_ or time for C_max_ was faster with NanoA (1.58 ± 1.28 h) and NanoB (1.08 ± 0.49 h), compared with the coconut oil solution (2.33 ± 0.82 h). The bioavailability values of NanoA and NanoB suspensions following oral administration were 28.6% and 22.7%, respectively, which were comparable to the coconut oil solution (28.4%), in which CCG-211790 had good solubility. 

## 4. Discussion

In our previous studies, we have identified a series of chemical compounds capable of inhibiting biofilm formation and virulence factor expression of *S. aureus* [6,9]. The poor aqueous solubility of the lead compound CCG-211790 has limited its therapeutic potential. In the current study, nanotechnology was applied to improve its pharmacokinetic properties for oral and intravenous delivery, which are common routes of delivery to prevent and treat wound infections. Two nanosuspension formulations (NanoA and NanoB) of CCG-211790 were generated using an antisolvent precipitation-ultrasonication method, a bottom-up approach. NanoA had a smaller particle size and different morphology in comparison with NanoB. The concentration of Tween 80 is a critical parameter in the determination of particle size and morphology of CCG-211790 suspensions. CCG-211790 suspensions of the z-average values from 290–350 nm could be reproducibly produced with the Tween 80 concentration less than 0.02% and the height of water filled in the ultrasonic tank from 10.4–29.5 mm. CCG-211790 suspensions of the z-average values from 500–1000 nm were obtained with the Tween concentration over 0.02% and the height of water from 8.1–9.0 mm. The size difference can be explained by the formation of Tween 80 micelles and the change of ultrasonic cavitation intensity with the height of the water. Critical micelle concentration (CMC) is defined as the concentration above which the discrete monomers of a surfactant start to form micelles, which is 0.0016% (*w/v*) for Tween 80. The formation of micelles increases the solubility of hydrophobic drugs by embedding drug molecules into the hydrophobic cores of the micelles. Therefore, Tween 80 micelles formed at a high concentration (e.g., 0.02% *w/v*) would dissolve and reduce the number of nuclei generated when mixing the organic solution with the antisolvent, which promotes the growth of particles. 

Ultrasonication and mechanical stirring also play important roles for reducing particle size. Ultrasonication can affect the diffusion coefficients of CCG-211790 molecules via the creation of acoustic cavitation [53]. Cavitation is a phenomenon in which a large number of small vacuum bubbles or voids are generated during the high-pressure cycles of ultrasonic waves and collapse during the low-pressure cycles releasing intense energy [54]. Cavitation is the major driving force for the collision or the fragmentation of existing crystals [55]. In our previous study, the hydrodynamic particle size (z-average) was reduced from over 600 nm to less than 400 nm when mechanical stirring from 100–600 rpm was applied during ultrasonication. Using mechanical stirring simultaneously with sonication, nanosuspensions of CCG-211790 with two different particle sizes, NanoA (315 ± 6 nm) and NanoB (915 ± 24 nm) were successfully generated. These nanosuspensions were demonstrated to be stable for six weeks.

**Figure 7:**
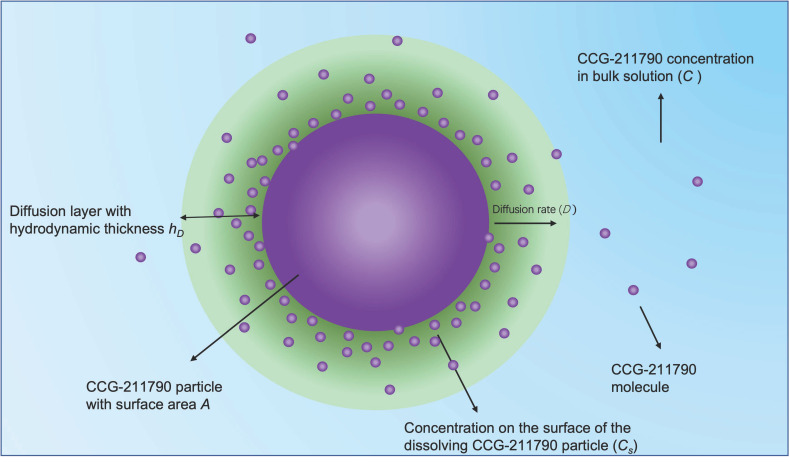
Schematic of the diffusion layer proposed by Noyes and Whitney and further modified by Nernst and Brunner. Particle size reduction leads to increased surface area A and decreased hydrodynamic layer thickness h_D_, therefore increasing dissolution rate dC/dt.

There was a significant increase in dissolution rates for NanoA and NanoB suspensions compared with CCG-211790 bulk powder. These higher dissolution rates can be explained by Noyes-Whitney equation: $\frac{\mathrm{dC}}{\mathrm{dt}}=\frac{\mathrm{DA}}{h_{D}}\cdot \left(C_{s}-C\right)$, where dC/dt is the dissolution rate of a drug from solid state, D is the diffusion coefficient of the drug in bulk solution, A is the effective surface area of the drug solid in contact with bulk solution, hD is the hydrodynamic diffusion layer thickness, C_s_ is the concentration on the surface of the dissolving solid and C is the concentration of the drug in bulk solution [56], as displayed in Figure 7.

Based on the fluid dynamic (FD) theory, the hydrodynamic diffusion layer thickness h_D_ can be described by Prandtl equation: $h_{D}=k\cdot \left(\frac{L^{1/2}}{V^{1/3}}\right)$, where L is the length of the surface in the direction of flow (equivalent to the diameter of particles), V is the relative velocity of the flowing liquid against a flat surface and k is a constant [57].

Though non-FD-based approximations to the hydrodynamic diffusion layer thickness have also been made by the Hintz-Johnson model and Wang-Flanagan model, it was mathematically shown that the FD model and non-FD model have similar h_D_ values for small particles with radii of less than 15 µm [58]. Particle size reduction leads to increased surface area A and decreased hydrodynamic layer thickness h_D_, thereby leading to increased dissolution rate based on Noyes-Whitney equation and Prandtl equation. Indeed, nanosuspension-based insoluble drugs may have sufficient dissolution rates for practical intravenous administration [59], and may even behave similarly to their solution formulations when the particle size is around 100 nm [60]. 

Although NanoB had larger dimensions in length and width, NanoB showed comparable dissolution rate to that of NanoA at every selected time point. NanoB consisted of plate- or tube-like particles with the thicknesses of plates or tube walls ranging between 100–300 nm (Figure 2C), which yielded a large surface area, thereby remarkably enhanced the dissolution rate. In comparison, CCG-211790 bulk powder displayed the slowest dissolution rate; 19.6% release of CCG-211790 in 5 min, 63.4% in 45 min and 64.3% in 60 min. The low dissolution rate can be explained by the small surface area and the poor wetting property of the CCG-211790 bulk powder [61]. Unlike CCG-211790 suspensions that immediately dispersed in the dissolution media, CCG-211790 bulk powder would disperse on the surface for 5–30 min before thoroughly immersing into the dissolution media when mild stirring (e.g., 75 rpm) was applied. In addition, the high standard deviation (9.9–20.7%) of CCG-211790 bulk powder dissolution rates at the early time points (from 5–30 min) can also be explained by the random divergence of wetting processes each time.

The pharmacokinetic behavior of drug nanoparticles is deeply associated with their dissolution properties. Drug nanoparticles that completely dissolve immediately after intravenous injection (typically with particles size of sub-200 nm) may have similar pharmacokinetic profiles to solution formulations [60,62]. In a pilot study of the PK properties of the powder suspension of CCG-211790, poor bioavailability was observed due to the poor aqueous solubility of the compound. The pilot study was conducted in feed animals (food not withheld), while the nanosuspension studies were conducted in fasted animals. A drug-food effect cannot be predicted on a scientific basis, requiring specific studies to understand possible effects [63,64,65]. However, the pilot study did demonstrate that the bulk compound had poor bioavailability which needed formulation technology to improve its PK properties.

NanoA and NanoB showed the trend toward lower C_max_ 0.8-fold and 0.66-fold respectively, as compared to PEG/PG solution. The lower C_max_ and faster clearance of the nanoparticle formulations may be due to CCG-211790 nanoparticles being slowly dissolved during the distribution phase. The in vitro dissolution study where complete dissolution of NanoA and NanoB suspensions occurred within 5 min after exposure to the dissolution medium indicated a rapid dissolution rate of CCG-211790 nanoparticles under a sink environment. An additional reason for the change in PK is possibly that CCG-211790 nanoparticles dissolved rapidly after reticuloendothelial system (RES) uptake leading to a better volume of distribution and high local tissue concentration. The high concentration in the liver contributed to the high elimination rate of the compound from the body via hepatic-metabolism, which may have reduced the concentration to lower levels. This assumption is supported by the higher clearances (CL) of NanoA and NanoB, which were 1.8-fold and 2.3-fold respectively to that of PEG/PG solution. This is possibly due to clearance mechanism of nanoparticles by macrophage/Kupffer cell-uptake in the liver [66]. The lower plasma concentration at all time points and the lower AUC (0.58-fold and 0.44-fold, respectively) of NanoA and NanoB suspensions compared to that of PEG/PG solution could also be explained by this hypothesis. Direct evidence of nanoparticle uptake in macrophage-rich organs can be determined through histological analysis, which can be conducted in future work. The V_z_s of the nanosuspensions were higher than PEG/PG solution, suggesting that the compound was well distributed into the body, and as an anti-virulence agent could act against the bacteria within the host tissues and organs for wound infection clearance and prevention of further complications such as systemic infections. 

The oral PK properties of the nanosuspensions were also studied to assess their potential as oral formulations because oral delivery is the most preferred route of drug delivery. The epithelia cells (e.g., intestinal epithelium) in the gastrointestinal (GI) tract function as a biological barrier that allow small molecules to pass through but block macromolecules or particles from entering systemic circulation [67]. Bioavailability of a water-insoluble drug can be enhanced by sufficient release of drug molecule from solid states or solubilizing vehicles into body fluids [68]. According to the Noyes-Whitney equation, the dissolution rate of drug nanoparticles can be significantly increased due to the large surface area, increasing GI uptake [69]. Furthermore, drug nanoparticles’ adhesion to biological mucosa in the GI tract can lead to accumulation on the GI mucosal surface enhancing retention for enhanced absorption [70]. While the C_max_s for all formulations were nearly the same as shown in Table 6 and Figure 6, the T_max_s or time for C_max_s were faster with the nanoparticle formulations and indicated that the drug would get into the blood and be distributed into the tissues faster, suggesting the nanosuspensions would have potentially faster onset of activity against the bacteria than solution formulations. Encouragingly, the bioavailability of NanoA or NanoB suspensions following oral administration was comparable with that of the coconut oil solution. Thus, converting water insoluble CCG-211790 into nanosuspensions could improve the PK properties of the compound to the levels of that of an oil-based solution.

In this study, PEG/PG solution was designed for intravenous administration. However, PEG/PG solution has no clinical potential because of limited solubility of powder CCG-211790 (up to 1 mg/mL) and the solution had poor storage stability with precipitation occurring within one day. Coconut oil can be used as the vehicle for oral formulations with the solubility of CCG-211790 up to 37 mg/mL [9] but is not suitable for intravenous administration. CCG-211790 nanosuspensions, particularly NanoA, have exhibited good storage stability, enhanced oral bioavailability comparable with coconut oil solution, good tissue distribution and the potential for both oral and intravenous administrations. Therefore, improved C_max_, tissue distribution and overall bioavailability for an anti-virulence agent could contribute to a clinical benefit for controlling bacteria without the need for oils or other excipients that may cause some tolerability/safety problems for oral administration and IV use.

Additionally, a drug candidate with poor aqueous solubility possesses low and highly variable oral bioavailability. As a result of the low oral bioavailability, the drug candidate must be administrated in a larger dose than required if it is to have higher bioavailability to achieve a therapeutically active concentration. Improving its bioavailability via nanosuspension approach could reduce the administered dose, thus decreasing the potential side-effects due to excessive drug dumping in the body, resulting in a lower cost of therapy [71,72,73].

The nanosuspensions can be formulated in diverse dosage forms to achieve higher loading capacities via different formulation methods such as wet milling, high-pressure homogenization, and nanocrystal powder-loaded tablets, which have the potential to further improve their bioavailability and exposure in tissues [33,34]. In addition, the pharmacokinetic properties of CCG-211790 nanosuspensions can be further optimized by surface modification with, for example, layer-by-layer assembled polyelectrolytes [74] or by coating with enteric polymers for controlled and extended release [75].

## 5. Conclusions

CCG-211790 nanosuspensions with different particle sizes and morphology, termed as NanoA (z-average of 315 ± 6 nm) and NanoB (z-average of 915 ± 24 nm), were produced using antisolvent precipitation-ultrasonication method. The majority of particles in NanoA were small particles with dimensions from 200–400 nm, while the dominant particles in NanoB were plate- or tube-like particles with lengths up to a few micrometers but thicknesses down to a hundred nanometers. Both NanoA and NanoB nanoparticles carried high magnitudes of negative zeta potential (up to 40–60 mV) and could maintain physical stability over 6 weeks when stored at 4 °C or room temperature. NanoA and NanoB suspensions exhibited markedly enhanced in vitro dissolution rates, with almost 100% release within 5 min and particle-like behaviors (e.g., lower C_max_s and larger V_z_s) during the distribution phase following intravenous administration in rats. NanoA and NanoB suspensions had oral bioavailability values of 28.6% and 22.7%, respectively. Bioavailability was comparable with that of the solution in a coconut oil formulation. Therefore, NanoA suspension is a promising formulation for the oral and intravenous administration for poorly soluble drugs, particularly for oral drug delivery, which is the most preferred administration route because it is noninvasive and can be self-administrated with high patient compliance, making it suitable for repeated and prolonged treatment outside a hospital setting. The pharmacokinetic properties can be further improved by using various approaches to enhance its potential therapeutic applications [76]. 

We have demonstrated the application of nanoparticle technology to improve the dissolution rate and overall bioavailability of a very insoluble antibacterial compound, CCG-211790 for treating wound infections. Using this approach in the future for drug candidates, it may be possible to generate products requiring lower doses, with reduced food effects, more rapid onset-of-action, and many other beneficial outcomes. Nanoparticles can target antimicrobial agents into tissues more efficiently. This can improve drug concentration at the site of infection, so that higher doses of the drug are achieved at the infected site, thereby potentially overcoming resistance. Additional nanoparticle-based strategies are providing designs for enhanced delivery of drugs for important therapies, including antibiotic-resistant bacteria in chronic wound infections and cancer [77,78,79,80]. Many of these formulation technologies are targeted delivery systems, directing the therapeutic agent directly to the target cells-bacteria/bacteria infected cells or cancer cells. The future of more effective and safer treatments for many diseases will be impacted by nanotechnology.

## Figures and Tables

**Table 1:** Stabilization of NanoA suspension storing at 4 °C or room temperature (RT) over six weeks.

Duration (Week)	Z-Average (nm)		Polydispersity Index		Zeta Potential (mV)	
	4 °C	RT	4 °C	RT	4 °C	RT
0	315 ± 6	315 ± 6	0.18 ± 0.03	0.18 ± 0.03	−42.3 ± 4.2	−42.3 ± 4.2
1	311 ± 1	310 ± 3	0.21 ± 0.01	0.22 ± 0.01	−49.3 ± 0.5	−48.4 ± 0.3
2	309 ± 3	316 ± 7	0.20 ± 0.03	0.20 ± 0.03	−49.4 ± 1.3	−49.5 ± 0.9
4	314 ± 2	322 ± 6	0.21 ± 0.01	0.23 ± 0.01	−46.1 ± 0.4	−52.3 ± 0.7
6	315 ± 3	319 ± 5	0.21 ± 0.01	0.20 ± 0.03	−45.4 ± 0.4	−51.3 ± 0.3

Each sample was measured in triplicate with mean values and standard deviations. No statistically significant difference was observed in comparison to their initial values (*p* > 0.05).

**Table 2:** Stabilization of NanoB suspension storing at 4 °C or room temperature over six weeks.

Duration (Week)	Z-Average (nm)		Polydispersity Index		Zeta Potential (mV)	
	4 °C	RT	4 °C	RT	4 °C	RT
0	915 ± 24	915 ± 24	0.52 ± 0.03	0.52 ± 0.03	−42.2 ± 0.2	−42.2 ± 0.2
1	900 ± 99	947 ± 60	0.58 ± 0.12	0.62 ± 0.02 *	−49.5 ± 0.5 *	−50.4 ± 0.7 *
2	911 ± 37	912 ± 66	0.61 ± 0.01 *	0.62 ± 0.07	−44.9 ± 0.6 *	−49.1 ± 0.7 *
4	966 ± 89	878 ± 17	0.59 ± 0.03	0.54 ± 0.04	−47.7 ± 1.0 *	−50.2 ± 0.3 *
6	856 ± 17 *	891 ± 24	0.57 ± 0.05	0.60 ± 0.05	−46.4 ± 1.4 *	−50.0 ± 1.5 *

Each sample was measured in triplicate with mean values and standard deviations. * *p* < 0.05, statistically significant difference was observed in comparison to their initial values.

**Table 3:** Pharmacokinetic parameters after intravenous administration of the DMSO/saline solution of CCG-211790 at a dose of 2.5 mg/kg in male Sprague–Dawley rats (*n* = 2).

PK Parameters	Pilot Study for IV
C_max_ (mg L^−1^)	1.21 ± 0.15
AUC_0∓∞_ (mg L^−1^ h)	0.67 ± 0.18
MRT_0∓∞_ (h)	0.95 ± 0.07
t_1/2β_ (h)	1.44 ± 0.15
V_z_ (L kg^−1^)	8.07 ± 2.92
CL (L h^−1^ kg^−1^)	3.85 ± 1.01

AUC: area under the concentration–time curve; C_max_: maximal concentration; MRT: mean residence time; t_1/2β_: terminal half-life; V_z_: volume of distribution during terminal phase; CL: clearance.

**Table 4:** Pharmacokinetic parameters of CCG-211790 powder suspension following oral administration at a dose of 5 mg/kg in male Sprague–Dawley rats (*n* = 2).

PK Parameters	Pilot Study for PO
C_max_ (mg L^−1^)	0.04 ± 0.01
T_max_ (h)	1.50 ± 0.71
AUC_0∓∞_ (mg L^−1^ h)	0.18 ± 0.03
MRT_0∓∞_ (h)	4.75 ± 1.20
t_1/2β_ (h)	3.88 ± 1.68
V_z_ /F (L kg^−1^)	168 ± 101
CL/F (L h^−1^ kg^−1^)	28.8 ± 5.6
F_IV_	13.4%

T_max_: time for C_max_; V_z_/F: apparent volume of distribution during terminal phase after non-intravenous administration; CL/F: apparent total clearance of the drug from plasma after oral administration; F_IV_: absolute bioavailability in regard to DMSO/saline solution following intravenous administration.

**Table 5:** PK parameters after intravenous administration of PEG/PG solution and NanoA and NanoB suspensions at a dose of 2.5 mg/kg in male Sprague–Dawley rats (*n* = 6).

PK Parameters	PEG/PG	NanoA	NanoB
C_max_ (mg L^−1^)	1.63 ± 0.25	1.31 ± 0.55	1.07 ± 0.44 *
AUC_0∓∞_ (mg L^−1^ h)	1.11 ± 0.14	0.64 ± 0.19 *	0.49 ± 0.12 *
MRT_0∓∞_ (h)	0.98 ± 0.17	0.90 ± 0.14	0.77 ± 0.21
t_1/2β_ (h)	1.13 ± 0.27	1.08 ± 0.18	1.06 ± 0.27
V_z_ (L kg^−1^)	3.66 ± 0.93	6.44 ± 1.84 *	8.37 ± 3.29 *
CL (L h^−1^ kg^−1^)	2.28 ± 0.27	4.21 ± 1.28 *	5.28 ± 1.01 *

* *p* < 0.05, statistically significant difference compared with PEG/PG solution.

**Table 6:** Pharmacokinetic parameters of the coconut oil solution (*n* = 6) and NanoA and NanoB suspensions (*n* = 6) following oral administration at a dose of 5 mg/kg in male Sprague–Dawley rats.

PK Parameters	Coconut Oil	NanoA	NanoB
C_max_ (mg L^−1^)	0.12 ± 0.07	0.13 ± 0.05	0.12 ± 0.03
T_max_ (h)	2.33 ± 0.82	1.58 ± 1.28	1.08 ± 0.49 *
AUC_0∓∞_ (mg L^−1^ h)	0.63 ± 0.27	0.63 ± 0.17	0.50 ± 0.08
MRT_0∓∞_ (h)	6.59 ± 3.22	5.00 ± 1.14	4.34 ± 1.03
t_1/2β_ (h)	3.82 ± 2.23	3.24 ± 1.36	2.61 ± 0.45
V_z_ /F (L kg^−1^)	46.1 ± 24.6	41.1 ± 21.7	38.2 ± 8.9
CL/F (L h^−1^ kg^−1^)	8.99 ± 3.14	8.53 ± 2.88	10.14 ± 1.64
F_PEG/PG_	28.4%	28.6%	22.7%

* *p* < 0.05, statistically significant difference compared with coconut oil solution.

## Data Availability

Data are contained within the article.
